# Solid Phase Peptide Synthesis on Chitosan Thin Films

**DOI:** 10.1021/acs.biomac.1c01155

**Published:** 2022-01-13

**Authors:** Tadeja Katan, Rupert Kargl, Tamilselvan Mohan, Tobias Steindorfer, Miran Mozetič, Janez Kovač, Karin Stana Kleinschek

**Affiliations:** † Institute of Chemistry and Technology of Biobased Systems (IBioSys), Graz University of Technology, Stremayrgasse 9, 8010 Graz, Austria; ‡ Department of Surface Engineering, Jožef Stefan Institute (IJS), Jamova 39, 1000 Ljubljana, Slovenia

## Abstract

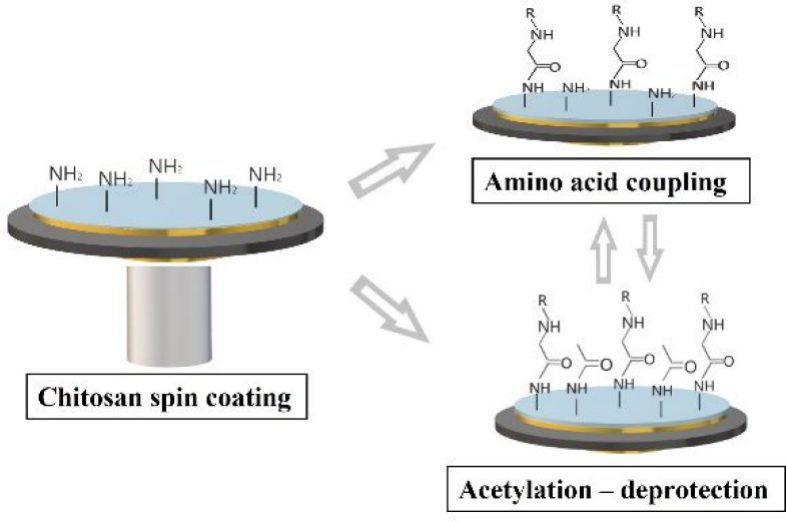

Stable chitosan thin films can be promising substrates for creating nanometric peptide-bound polyglucosamine layers. Those are of scientific interest since they can have certain structural similarities to bacterial peptidoglycans. Such films were deposited by spin coating from chitosan solutions and modified by acetylation and *N*-protected amino acids. The masses of deposited materials and their stability in aqueous solutions at different pH values and water interaction were determined with a quartz crystal microbalance with dissipation (QCM-D). The evolution of the surface composition was followed by X-ray photoelectron (XPS) and attenuated total reflectance infrared (ATR-IR) spectroscopy. Morphological changes were measured by atomic force microscopy (AFM), while the surface wettability was monitored by by static water contact angle measurements. The combination of the characterization techniques enabled an insight into the surface chemistry for each treatment step and confirmed the acetylation and coupling of *N*-protected glycine peptides. The developed procedures are seen as first steps toward preparing thin layers of acetylated chitin, potentially imitating the nanometric peptide substituted glycan layers found in bacterial cell walls.

## 1 Introduction

The backbone of peptidoglycans is an alternating copolymer of β-1-4 linked *N*-acetyl-d-glycosamine (GlcNAc) and *N*-acetyl D-muramic acid (MurNAc) and comprises the main dry mass of most bacterial cell walls arranged in nanometric layers.^1,2^ Peptidoglycans can be viewed as a type of chitin derivative, or poly β-1–4 *N*-acetyl d-glucosamine. Deacetylated chitin or chitosan could therefore be a potential precursor if one attempts to semisynthetically mimic the structure of peptidoglycans.^3^ Such mimetics can be of interest for studying phenomena at interfaces such as adsorption,^4^ enzymatic digestion,^5^ live-cell adhesion,^6^ or lithography.^7,8^ A defined semisynthetic peptidoglycan coating could also contribute to insights into biological pattern recognition,^9^ cell–cell communication,^10,11^ or immune response^12^ and can increase our understanding of bacteria.^13^ Peptidoglycans are however very complex, and bacterial cell walls do not only consist of polysaccharides and peptides. The first steps toward a potential mimetic based on chitosan would therefore rely on investigating conditions for preparing thin coatings, followed by acetylation into some form of chitin, and derivatization with suitable peptides. Thin film formation could potentially also be performed after derivatization,^14^ but chitin films are more tedious to prepare due to the limited solubility of the precursor, requiring either derivatization^15^ or direct dissolution prior to coating.^16^ A heterogeneous derivatization as a preformed thin film coating could therefore be an attractive alternative.^17^ Thin chitosan coatings have traditionally been prepared by electrodeposition^18^ or adsorption^19^ from aqueous solutions, followed by chemical modification.^20,21^ Spin coating has first been reported for waveguides.^22^ Other works included coatings of silicon wafers with melamine resins,^23^ coating of glass,^24^ and implant materials^25^ for sensors^26^ or as lipid bilayer supports.^27^ Though many studies make use of chitosan as a solid support for other materials, basic reports on the spin coating of pristine chitosan without additives are more limited.^8,28^ Works on acylation of spin-coated thin films to obtain a type of acetyl-chitin was suggested but not performed by Cheng et al.^8^ Murray et al. report on the thermal reaction of chitosonium acetate thin films as an alternative to acetylation with anhydrides, but the high temperatures necessary lead to partial film distruction.^28^ Otherwise, direct acetylation of thin films with, for example, acetic anhydride, could not be encountered to the best of our knowledge in the literature.^29−32^ Regarding the immobilization of peptides, Neugebauer et al. developed a method for the solid-phase peptide synthesis (SPPS) on chitin powders in organic solvents followed by capping of unreacted groups by acetic anhydride and cleavage of the peptide.^33^ Meerovich et al. used chitosan particles for SPPS also with a final cleavage of the desired peptide.^34^ There are only few reports on the binding of finished peptides to chitosan after film formation. Costa et al. immobilized antimicrobial peptides on chitosan thin films using NHS esters of cysteine or polyethylene glycol.^35^ Similarly, Monteiro et al. used maleimides.^36^ Other authors studied protein^37−39^ or antibody interaction in or on composite films^40^ and Barbosa et al. reported about click chemistry for peptides on chitosan powders.^41^ To develop the field further, this work aims at elaborating and combining film acylation into a type of acetyl-chitin with the solid phase peptide synthesis on these films. This should pave the way toward possible peptidoglycan mimetics but can also find other applications. After spin coating, the materials are characterized by infrared (ATR-IR) and X-ray photoelectron spectroscopy (XPS), atomic force microscopy (AFM), and water contact angle measurements. Subsequently, the films are neutralized and chemically derivatized by acetylation to produce a type of chitin mimetic otherwise not accessible. The dry mass deposition, pH, and solvent stability are assessed in the microgram regime by a quartz crystal microbalance with dissipation monitoring (QCM-D). Binding of *N*-protected amino acids using carbodiimide chemistry in water and DMSO including deprotection is then studied by XPS, AFM, ATR-IR, and QCM-D on silicon or gold-coated surfaces.

## 2 Experimental Section

### 2.1 Materials

Chitosan CH (75–85% deacetylation, low molecular weight), Boc-glycine Boc-Gly-OH, Fmoc-glycine Fmoc-Gly-OH, *N*-(3-(dimethylamino)propyl)-*N′*-ethyl-carbodiimide-hydrochloric (EDC hydrochloride), and 1-hydroxybenzotriazole hydrate (wetted with not less than 14 wt % water, 97%) were purchased from Sigma-Aldrich, Austria. Hydrochloric acid (37%), sodium hydroxide, and ammonia (25%) were purchased from VWR Chemicals. Dimethyl sulfoxide DMSO (≥99%) was purchased from Fluka Chemicals. Hydrogen peroxide (30%, for synthesis) and triethylamine, TEA (≥99.5%, for synthesis), were purchased from Carl Roth, Germany. Sulfuric acid S.G. 1.83 (>95%) was purchased from Fisher Scientific, Austria. Silicon wafers with orientation ⟨100⟩, resistivity 15.00 Ohm cm, and thickness of 508 μm were purchased from Silchem, Freiburg, Germany. QCM-D gold-coated sensors QSX301 were purchased from Quantum Design Europe, Darmstadt, Germany.

### 2.2 Substrate Cleaning

Thin films of chitosan were deposited on two types of substrates, either 15 × 15 mm^2^ silicon wafers or QCM-D gold-coated substrates. To make the silicon wafer surface more hydrophilic, they were immersed in a solution of hydrogen peroxide (30% in water) for 1 h, then immersed into Milli-Q water for 15 min. Each substrate was then extensively washed with deionized water, and potential residues were cleaned with a cotton swab and water and dried with nitrogen gas. QCM-D sensors were immersed into a solution consisting of H_2_O/H_2_O_2_ (30%)/NH_4_OH (25%) in a ratio 5:1:1 (v/v/v) for 15 min at 70 °C, followed by “piranha” solution containing H_2_O_2_ (30%) and H_2_SO_4_ in ratio 1:3 (v/v) for 45 s.^42^ Special care has to be taken when using piranha solutions since the reaction is extremely exothermic and the solution is very corrosive, especially to biological tissue. In between piranha solutions, samples were dipped into Milli-Q water for 15 min and stored in Milli-Q water. Finally, each sensor was cleaned with Milli-Q water, and all potential residues were removed with a cotton swab soaked with acetone and blown dry with nitrogen gas.

### 2.3 Chitosan Solution

Chitosan solution at a concentration of 1 wt % was prepared by adding the polymer powder to water and adjusting to a pH value of 2 with 0.1 M HCl. The solution was left to mix for 1 h, then 0.1 M NaOH was added dropwise while stirring until pH 5 was reached. A PTFE syringe filter was used (pore size 1 μm) to filter the produced solution three times before storing it.

### 2.4 Spin Coating

A Spin200i spin coater (SPS Europe) was used to produce uniform thin chitosan films on silicon wafers and on gold-coated QCM-D sensors. The amount of applied solution was 25 μL. The program used for spin coating consisted of three steps; a surface wetting step with a slow rotation of 10 rpm for 10 s, solution spreading step for 30 s at 5000 rpm, and a drying step for 30 s at 2000 rpm.

### 2.5 Thin Film Stabilization

To stabilize the chitosan thin films in water, they were immersed into 5 mL 0.5 M NaOH for 10 min followed by extensive drying with nitrogen gas before immersing them for 10 min into Milli-Q water and drying them again. This procedure causes neutralization and thus stabilization.^43^ Neutralization experiments were also conducted with 0.1 M NaOH under the same conditions.

### 2.6 Chitosan Acetylation

Acetylation of as-deposited chitosan thin films was performed both on silicon wafers and QCM gold-coated sensors. The reaction was carried out at room temperature with 5 mL of pyridine/acetic anhydride 1:1 (v/v) mixture into which the samples were immersed for 1 h. This was followed by soaking in DMSO and Milli-Q water each for 15 min and dried with nitrogen gas.

### 2.7 Amino Acid Coupling

Coupling was done with an excess amount, that is, 0.2 mmol (to mol available nitrogen atoms on film), of commercial N-protected glycine with an equimolar amount of EDC coupling reagent in the presence of a base. Boc-Gly-OH solution (4 mM) was prepared in 50 mL water, and sodium hydroxide was used to adjust to pH 9. Fmoc-Gly-OH (4 mM) was dissolved 50 mL of DMSO, and 72 μL of triethylamine was used with an equimolar amount of HOBt to EDC. In both cases, the films were incubated for 3 h before treating with DMSO, water washing, and nitrogen drying. For Fmoc deprotection of glycine, films were incubated for 1 h in 20% piperidine in DMSO, followed by the repetition of Fmoc-Gly coupling as described before.

### 2.8 Analytical Methods

#### 2.8.1 X-ray Photoelectron Spectroscopy (XPS)

Films deposited on silicon wafers or gold-coated crystals were subjected to X-ray photoelectron spectroscopy (XPS) measurements. Analysis was carried out on a PHI-TFA XPS spectrometer produced by Physical Electronics Inc. and equipped with monochromatic Al source. The analyzed area was 0.4 mm in diameter. The sampling depth was estimated from the relationship *d* = 3λ_AL_ sin α, where *d* is the sampling depth in nanometers, λ_AL_ is the attenuation depth, which is energy dependent and element specific. α is an emission angle with respect to the surface, which is in our case 45°. Typical values for λ_AL_ are for organic materials around 3.0 nm for C 1s photoelectrons at kinetic energy 1200 eV (corresponding to binding energy 285 eV). In this way, sampling depth for electrons of interest C 1s, N 1s, O 1s for organic material was estimated to be in the range between 5 and 7 nm.^44^

The high-energy resolution spectra were acquired with the energy analyzer operating at a resolution of about 0.6 eV and pass energy of 29 eV. During data processing, the spectra were aligned by setting the C 1s peak at 286.3 eV, characteristic of C–OH bonds present in chitosan. The accuracy of binding energies was about ±0.3 eV. Quantification of surface composition was performed from XPS peak intensities considering relative sensitivity factors provided by the instrument manufacturer.^45^ Two places on every sample were analyzed and average composition was calculated. High-resolution spectra were fitted with Gauss-Lorentz functions, and Shirley function was used for background removal.

High-energy resolution XPS spectra were fitted with model peaks, which are characteristic for specific bonds of C, O, and N atoms. In this way C–C/C–H, C–OH/C–N, O–C–O/C=O, O–C=O bonds were identified in the C 1s spectra, C–NH_2_, C–NH_3_ bonds in the N 1s spectra and CH_2_–OH, C=O bonds in O 1 s spectra. The assignment of the identified peaks by fitting procedure might not be unique but it is commonly used in XPS analyses of organic materials. The change of relative concentration of the specific bond was explained for each step of film treatment in discussion.

#### 2.8.2 Time-of-Flight Secondary Ion Mass Spectrometry (ToF-SIMS)

Time of flight secondary ion mass spectrometry (ToF-SIMS) was performed using a ToF–SIMS 5 instrument (ION-TOF, Münster, Germany). A Bi_3_^+^ cluster ion beam with a kinetic energy of 30 keV was used as an analytical beam.

#### 2.8.3 Attenuated Total Reflectance-Infrared Spectroscopy (ATR-IR)

Infrared spectra were measured using ALPHA-P, Bruker spectrometer. Scan range was set from 4000 to 375 cm^–1,^ with a total of 40 scans performed at a resolution of 4 cm^–1^. Only gold-coated QCM crystals were used as substrates.

#### 2.8.4 Atomic Force Microscopy (AFM)

Morphology was investigated with atomic force microscope Tosca 400, Anton Paar (Graz, Austria). The images were scanned in tapping mode with silicon SPM-Sensor (Arrow-NCR-50, Nanoworld, Switzerland) with a resonance frequency of 285 kHz and a force constant of 42 N/m. AFM images were acquired for thin films deposited either on silicon substrates or gold-coated quartz crystals. Image sizes of 10 μm × 10 μm, 5 μm × 5 μm, and 1 μm × 1 μm were scanned at a speed of 0.9 lines per second. Measurements were performed at room temperature. Image processing was done using the Gwyddion program.^46^

#### 2.8.5 Profilometry

The thickness of the film deposited on silicon substrates was determined using Dektak XT, Bruker. The scan profile was set to hills and valleys, and scan length was 2000 μm in 10 s. Stylus radius was 12.5 μm with a force of 3 mg and resolution of 0.666 μm/pt. In order to determine the film thickness, the surface was scratched with a small razor blade to remove the deposited film and obtain film heights using a step-height profile. Film thickness was determined at three different positions on one substrate, and a standard deviation was calculated.

#### 2.8.6 Contact Angle

In order to determine the wettability of thin film surfaces, contact angle measurements were performed. Using a Drop Shape Analyzer (DSA 100, Krüss), a static contact angle was measured. Measurements were acquired at room temperature with a droplet volume of 2 μL. At least three different measurements were performed for each sample, and an average with standard deviation was calculated. The water contact angle on chitosan samples was measured on three different occasions to determine how wettability changed with film aging. For this purpose, measurements on day 0 (right after the film was made), day 7, and day 14 were made. For each measurement, a new sample was used, and no sample was used twice. In between measurements, films were stored in a desiccator under a vacuum with calcium chloride as a drying agent.

#### 2.8.7 Quartz Crystal Microbalance with Dissipation (QCM-D)

A QCM-D instrument (model E4) from Q-Sense (Gothenburg, Sweden) was used. The instrument simultaneously measures changes in the resonance frequency (Δ*f*) and energy dissipation (Δ*D*) when the mass of an oscillating piezoelectric crystal changes due to the removal/deposition of material. Dissipation refers to the frictional losses that lead to damping of the oscillation, which depends on the viscoelastic properties of the material. For a rigid adsorbed layer that is fully coupled to the oscillation of the crystal, Δ*f*_*n*_ is given by the Sauerbrey equation. A detailed explanation of the technique can be found elsewhere.^47^

The frequency of individual crystals was measured in air for an uncoated sample, after chitosan spin coating, after neutralization, and after acetylation or amino acid coupling. The frequency of the measured crystals was stitched together using QSoft 401 software (Biolin Scientific) to compare frequency shifts and thus deduce the mass changes. The stability of all coated films in water and acidic and alkaline media was studied *in situ* by pumping the solutions through the flow chambers at a flow rate of 0.1 mL/min and at room temperature.

The water content in chitosan thin films studied with H_2_O/D_2_O exchange experiment, as initially reported by Kittle et al.^48,49^ Films were preswelled with a flow rate of 0.1 mL/min in Milli-Q-water for 2 h before the experiment, followed by a 10 min rinse with D_2_O and last 10 min rinse with Milli-Q water. For water content calculations, 0.9982 g/cm^3^ density for H_2_O and 1.1050 g/cm^3^ density for D_2_O were used with the Sauerbrey constant *C* of −0.0177 μg/cm^2^ for a 5 MHz QCM crystal.

## 3 Results and Discussion

### 3.1 Composition of Thin Films

**Figure 1:**
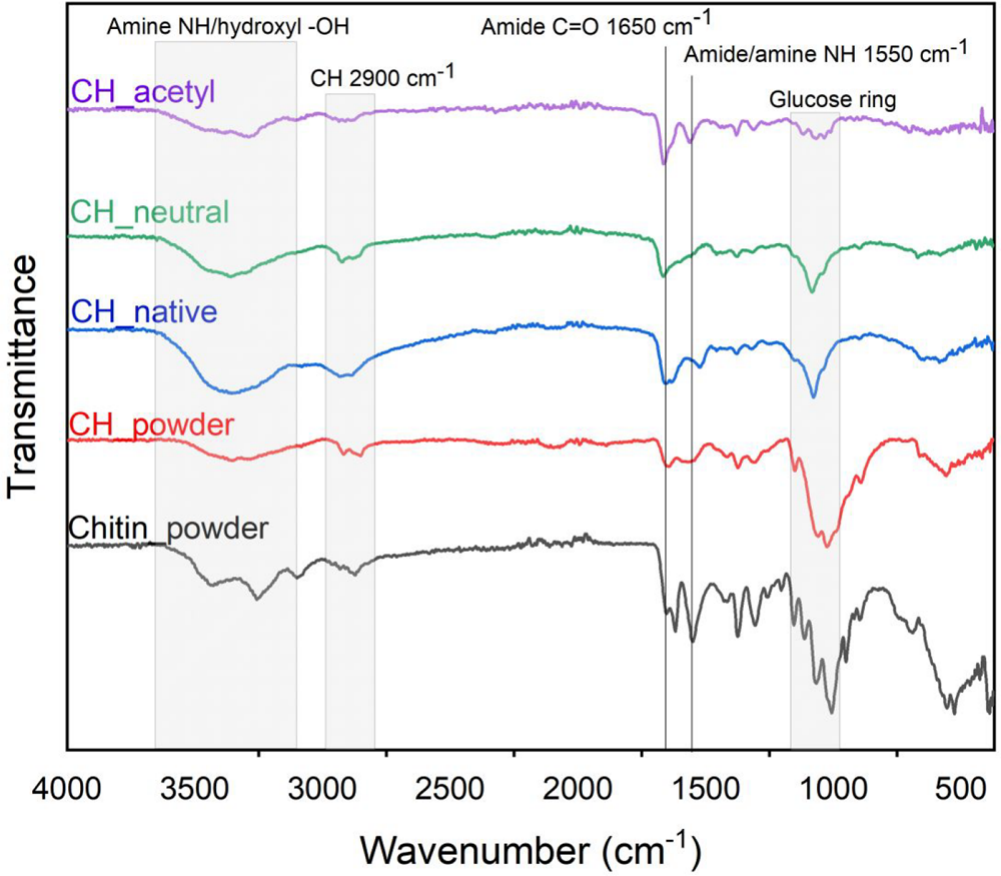
ATR-IR spectra of chitin and chitosan powder, chitosan thin films in native, neutralized, and acetylated form.

Figure 1 shows the ATR-IR spectra of a chitosan thin film (CH_native) in its native and neutralized form (CH_neutral). Among its characteristic peaks of protonated NH_3_^+^ and −OH stretch band in the region of 3600 to 3100 cm^–1^, which cannot easily be distinguished due to overlaying, there are also some other major changes owing to amide stretching. Deprotonation of amine moiety is observed with changes in the region of 1700–1500 cm^–1^ specifying C=O amide stretching and N–H amine bending. The peak at 1530 cm^–1^ of the native chitosan area assigned to N–H bending is reduced and shifted to higher wavenumbers (1600 cm^–1^) due to amine group deprotonation.^50^ On the other hand, no shift is observed in the C=O amide stretch at 1700 cm^–1^, indicating no changes of the acetylated part of chitosan (CH_neutral) after deprotonation. Furthermore, a slight shift of sugar ring at 1000 cm^–1^ is detected in films as opposed to powder. A peak at 1310 cm^–1^ assigned to the amino group is detected in all spectra as well as glycosidic C–O stretching at 1080 cm^–1^. These results align with literature reviews of macroscopic chitosan films.^50^ In the acetylated samples, a visible decrease in OH broad peak at 3400 cm^–1^ suggests that hydroxyl groups were esterified. A peak of the acetyl C=O at a wavenumber of 1660 cm^–1^ is visible in (CH_acetyl) as opposed to neutralized chitosan film, pointing toward successful N-acetylation and ester formation. The spectra of CH_acetyl do show similarities with the spectra of chitin powder. In general, amine groups are considered more nucleophilic than hydroxyl groups under the applied conditions, and a majority of amine acetylated products would be obtained. Apart from those, additional small peaks, specifically the 3100 cm^–1^ peak for C–H stretching and 1400 cm^–1^ for C–H bending, show the presence of alkane groups.

**Figure 2:**
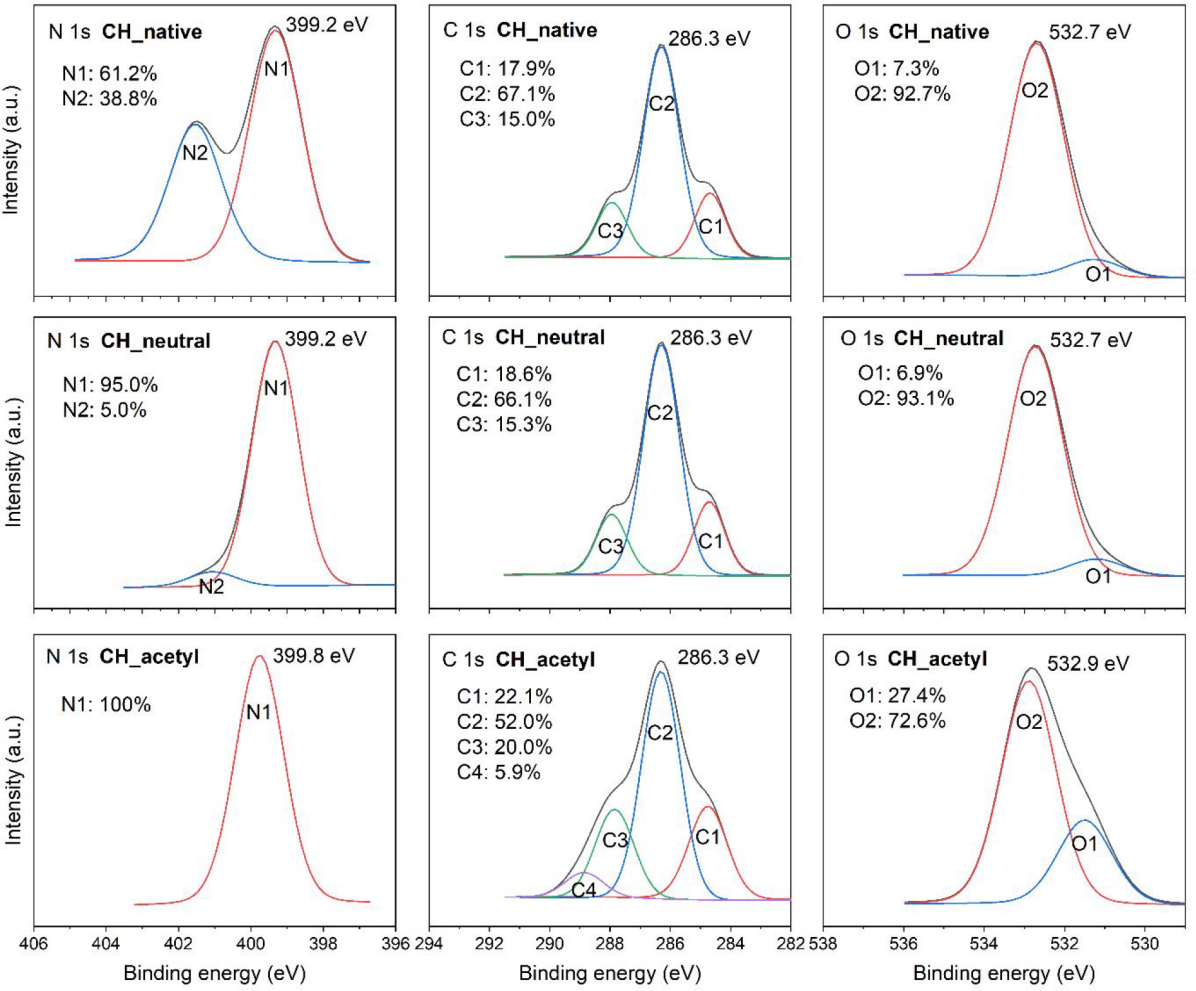
XPS high-energy resolution spectra N 1s, C 1s and O 1s on native chitosan (CH_native), neutralized chitosan (CH_neutral), and acetylated chitosan (CH_acetyl) thin-film samples.

Surface elemental composition and bonding modes of chitosan native and neutralized thin films were further analyzed by XPS, which gave us a valuable insight into the deprotonation of chitosan. The surface elemental composition of produced films was measured on silicon wafers, and the results are gathered in Table S1 (see Supporting Information). The theoretical composition of glucosamine is 54.5 atom % carbon, 36.4 atom % oxygen, and 9.1 atom % nitrogen with oxygen to carbon O/C ratio of 0.67 in contrast to 0.57 O/C ratio of actual samples of chitosan films. For the reason that XPS is a very surface-sensitive technique, one must account for the higher atomic composition of C, O, and consequently lower O/C ratio in actual samples compared to theoretical calculations due to contaminations. Besides the atomic composition of chitosan, not much about deprotonation could be extracted from elemental composition data (wide energy spectra Figure S3). However, the high-resolution XPS spectra of nitrogen N 1s reveal significant changes, so nitrogen deprotonation is detected. Before treatment with sodium hydroxide, two types of nitrogen bonds were present, namely C–NH_2_ (399.2 eV)^51^ and C–NH_3_^+^ (401.3 eV) with 61% and 39% of relative concentration, respectively (Figure 2). However, after the sodium hydroxide treatment 95% C–NH_2_ bond type is detected. This result suggests that the procedure for neutralization with sodium hydroxide was successful and that predominantly deprotonated chitosan is present afterward. For acetylated samples, it is evident that the elemental composition of carbon, nitrogen, and oxygen align with theoretical values. The actual ratio trend of oxygen to carbon O/C is comparable to theoretical calculations and is higher on neutralized surfaces as opposed to acetylated chitosan surfaces due to two added carbon atoms as opposed to one oxygen atom thus making the ratio smaller.

High-energy resolution XPS spectra of carbon C 1s were deconvoluted into different peaks related to different bonds of carbon atoms. Peaks represented as C2 at 286.3 eV (Figure 2) are attributed to C–O (hydroxyl) or C–N bond and are similar to those calculated theoretically and reported elsewhere.^51^ In addition, a C3 peak at 287.9 eV belonging to the O–C–O bond also matches theoretical calculations of pure chitosan by Kostov et al.^51^ However, an additional peak C1 related with C–C/C-H bonds at 284.7 eV is seen, which should be absent in pure chitosan, so this is attributed to hydrocarbons adsorbed onto the sample surface. The same peak is normally also seen in cellulose when probed by XPS.^51,52^ Carbon C 1s XPS spectra before and after capping show several differences. First, an additional peak, namely C4 at 288.9 eV (Figure 2), can be detected after acetylation with an area of 5.9% of the total intensity of carbon spectrum C 1s. It represents the O=C–O carbon bonds which are most likely due to O-acetylation of hydroxyl groups on the third or even more likely sixth chitosan carbon atom. Second, C1 at 284.7 eV, C2 at 286.3 eV, and C3 at 287.9 eV peak areas of both neutralized and acetylated samples, normalized to 100%, give the ratios of C1/C2/C3 before capping 1:5:1 and after capping 1:3:1. Ratios clearly indicate a decrease in C2 binding type attributed to C–OH and/or C–N bonds, suggesting some amount of O-acetylation and consequently decrease in C2 peak area relative to the C3 peak area (O/N acetyl), besides that also an increase in ratios of C–H bonds (C1) can cause a shift in this ratio.

Oxygen atoms of a pure chitosan structure are found in four distinguished bonds. One is allocated in glycosidic bond, one is incorporated in the ring, and two are in hydroxyl groups, both bonded to the CH_2_ group. Both glycosidic O atoms and the one in the ring as well as −CH_2_OH bond have a similar theoretical binding energy of 535 eV in the oxygen O 1s XPS spectrum and contribute to the O2 peak with the higher area, observed in Figure 2 at 532.8 eV. These results are similar to experimental conclusions reported in other literature.^51^ The hydroxyl group bonded directly on the glucose ring is usually experimentally depicted with a peak binding energy of around 531.7 eV. Furthermore, confirmation of successful acetylation is clearer in O 1s XPS spectra. Here, an increase in O1 peak area at 531.5 eV after capping, assigned to C=O bonds, confirms the acetylation of the chitosan surface. To obtain a pure chitin surface, N-acetylation would be preferable. However, according to the C 1s spectrum, O-acetylation should not be ruled out.

### 3.2 Mass, Thickness, Wetting, and Morphology of Chitosan Films

**Figure 3:**
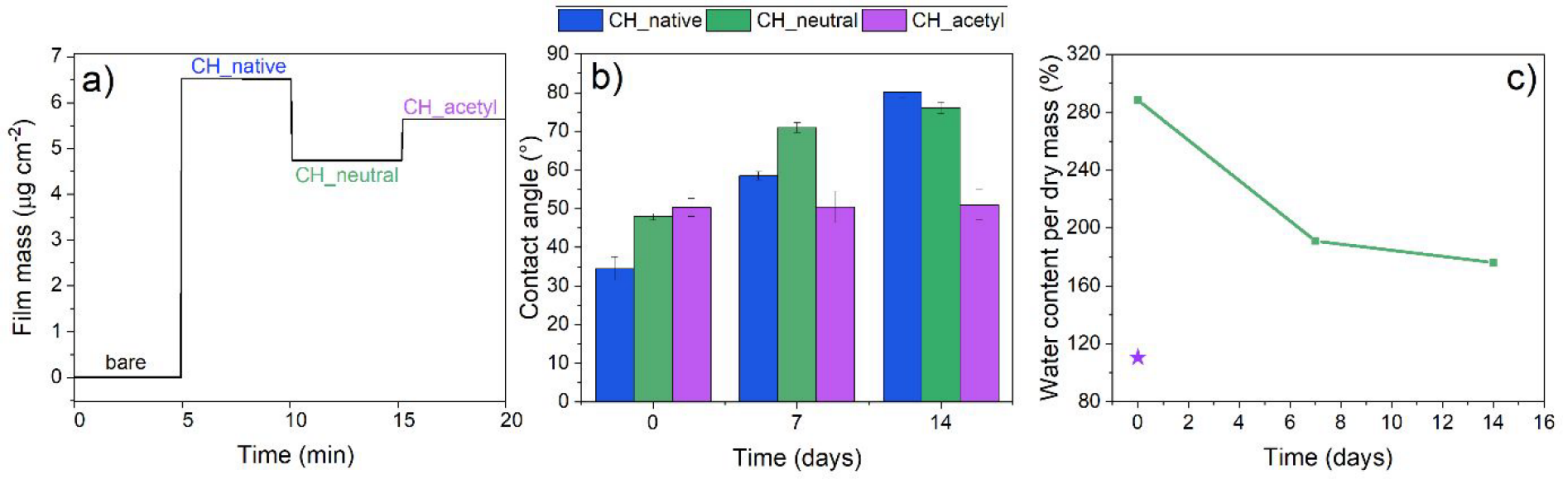
(a) Stitched QCM-D data of dry film mass for native (CH_native), neutralized (CH_neutral), and acetylated (CH_acetyl) chitosan thin films (coatings and derivatizations were performed in-between each QCM-D measurement step). (b) Water contact angle measurements of aged native (CH_native), neutralized (CH_neutral), and acetylated (CH_acetyl) chitosan thin films over 14 days. (c) Calculated water content determined from D_2_O/H_2_O solvent exchange of aged, neutralized chitosan thin films (CH_neutral) over 14 days stored in desiccator, green, compared to acetylated (CH_acetyl) samples at day 0, purple.

By QCM-D, mass changes as small as 17.7 ng/cm^2^ can be measured as well as changes in film swelling according to its surroundings.^19^ Here, a baseline with a bare gold-coated crystal was established (0 Hz), following by spin-coated chitosan solution giving a frequency shift to −368 Hz and last, after neutralization, the frequency rises to −267 Hz (Figure 3a). Spinning the chitosan solution onto gold-coated QCM crystals yielded a film mass of 6.51 μg/cm^2^, corresponding to the frequency of −368 Hz. Nevertheless, after neutralization the mass decreased by about 25%, giving a final film with 4.6 ± 0.35 μg/cm^2^ mass observed. It is assumed that during the neutralization, some parts of the upper chitosan layer are desorbed. With profilometry measured heights of thin film deposited on a silicon wafer for CH_native is 43.5 ± 1 nm and of CH_neutral 32 ± 3 nm, indicating a 26% height loss, supporting the QCM-D data and indicating that the effect is not shrinkage but desorption (Figure 3a). Special care was taken to get reproducible results of mass deposited and the desorption effect can also be considered reproducible. Finally, these films possess sufficient stability after neutralization to allow for a subsequent chemical derivatization. We paid attention to exact incubation times in alkaline solution and water as well as extensive drying with nitrogen gas in-between, to wash all NaCl salt from the film surface. When related to the chitosan film mass (Figure 3a), a theoretically full acetylation of all amines would cause a mass increase of 1.2 μg/cm^2^. The actual weight after capping is 5.4 ± 0.30 μg/cm^2^, a difference of 0.8 μg/cm^2^ making the acetylation successful but not complete, but resulting into an insoluble acetyl chitin thin film. It is however important to note that film leaching during capping could underestimate the degree of acetylation.

Static water contact angles of CH_native could only be measured within the first 3 s of droplet contact, since the surfaces are soluble and hydrophilic leading to the spreading of the drop. CH_neutral and CH_acetyl showed stable contact angles after drop deposition. Several papers of macroscopic chitosan films reported very high contact angles of about 95°.^53,54^ Also in our case CH_neutral and CH_acetyl gave higher water contact angle values than the native form but both cannot be considered hydrophic surfaces.

The results show that the contact angles change considerably after aging for all surfaces except CH_acetyl (Figure 3b). This can be explaned by structural rearrangements and water desorption upon drying during which new hydrogen bonds could be formed and hydrophobic parts move toward the air interface. Such phenomena are described for cellulose thin films and known as hornification.^55^ This effect could also be confirmed by a reduced water content of aged versus pristine CH_neutral films (Figure 3c) as determined by D_2_O/H_2_O exchange. The water absorption of pristine CH_neutral is almost three times their dry mass, whereas this is reduced to two times the dry mass of aged samples. This supports the hypothesis of an irreversible drying and rearrangement. On the contrary, CH_acetyl has a significantly lower water content (1.5 times the dry mass) and a stable contact angle over time.

**Figure 4:**
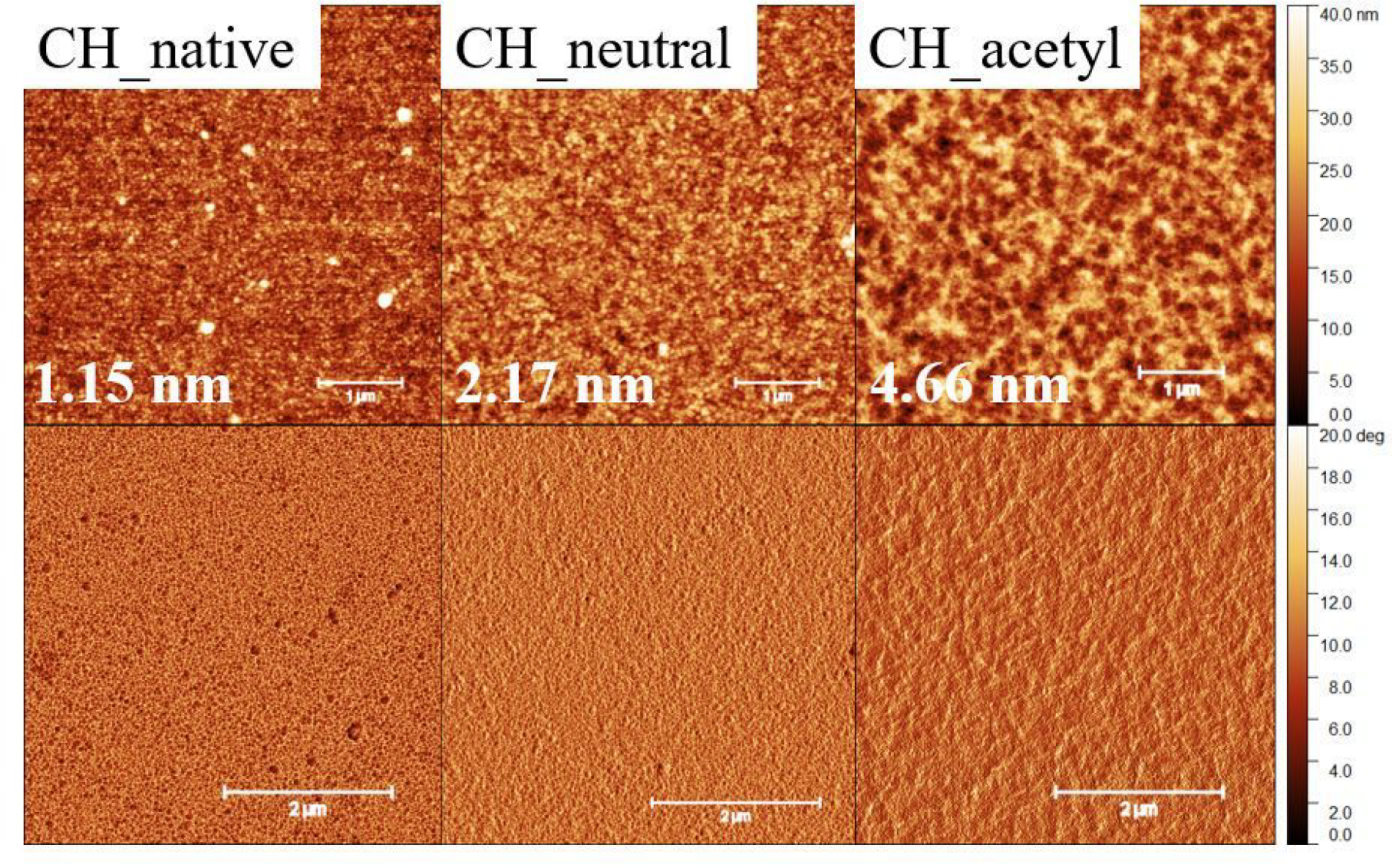
AFM images (5 × 5 μm^2^). TOP row topography, BOTTOM row phase image: chitosan native thin film (CH_native), chitosan neutralized thin film (CH_neutral), and acetylated chitosan (CH_acetyl). RMS roughness values are shown for the depicted height images.

AFM height and phase images for 5 × 5 μm^2^ (Figure 4) show a significant increase in RMS roughness upon neutralization (RMS roughness, CH_native 1.1 ± 0.1 nm; CH_neutral 2.35 ± 0.2 nm). Acetylation causes an even higher increase of RMS roughness (CH_acetyl 4.66 nm). However, all images confirm the absence of larger defects and the presence of intact coatings at the area investigated.

### 3.3 Film Stability in Aqueous Environments

**Figure 5:**
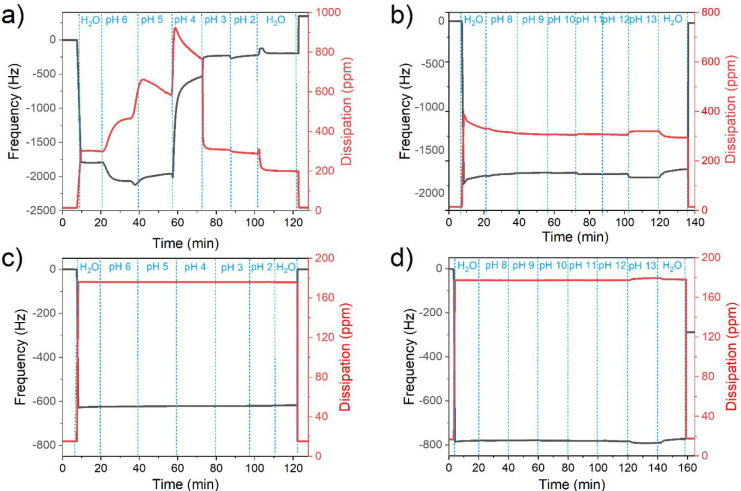
Realtime QCM-D measurements of CH_neutral in (a) acidic and (b) alkaline, and of CH_acetyl in (c) acidic and (d) alkali environment. After rinsing with water and drying, the remaining dry mass values are shown and can be compared with the initial value measured before exposure to liquids.

Figure 5 shows the frequency and dissipation changes upon conditions in which CH_neutral or CH_acetyl films were gradually exposed to water with decreasing or increasing pH values. At the introduction of Milli-Q water, after air exposure the frequencies to −1800 Hz and dissipation to 270 ppm are shifted (Figure 5a). This is a consequence of the higher density of water. In this case, the measurements were stopped and the films were taken out and N_2_ dried after this step; the pristine and final dry masses were not changed confirming that the film stayed intact throughout the rinsing with neutral water. The same was observed for the dissipation values (data not shown). When measurements were continued uninterrupted with decreasing pH 6, an intense swelling was observed, but films remained stable. It is interesting to see that already this pH value seems to cause a considerable protonation of the amine in the film leading to swelling. However, at the borderline between pH 5 to pH 4, one can observe a sharp increase toward higher absolute frequency values. This stands for a sudden decrease in mass, which indicates partial detachment of the material due to dissolution. Protonation of the chitosan (p*K*_a_ 6 primary amine^20^) and rapid removal of the thin film shift the frequency from −1900 to −700 Hz. As the film is being protonated, the increasing internal charge repulsion between neighboring protonated polybasic groups is rising and causes strong hydration with dissipation changing from 800 to 300 ppm as the pH changes from 4 to 3. After the tipping point at pH 4, the majority of dissolved chitosan was flushed away, still leaving some covering. It is apparent from Figure 5a, that the end dry mass of the substrate is lower than the starting mass since the frequency shift is positive (about 600 Hz).

AFM images of the surfaces rinsed at pH 5, pH 4, and pH 3 are shown in Supporting Information (Figure S1). The RMS roughness of the chitosan surface changes slightly from 2.25 nm at pH 5 to 2.33 nm at pH 4. Furthermore, a more visible change is detected at pH 3 with a roughness decrease to 1.70 nm, confirming some disintegration of the chitosan film.

Figure 5b shows the frequency and dissipation upon exposure of CH_neutral films to alkaline solutions. Except for the initial shift from air to water (at the time of about 8 min), both frequency and dissipation values remain stable up to pH 12. At pH 13, an instant increase in dissipation and decrease in frequency was detected. AFM images acquired for such samples reveal a rougher surface (Figure S1). It is known that alkaline solutions are used for the swelling of cellulose, and similar mechanisms can also explain the observed phenomena of chitosan swelling. An explanation would be the presence of alkoxide RO^–^ in the chitosan structure and intercalation of sodium or hydroxyl ions together with stronger water interactions. The dry film mass did not change as compared to the initial dry mass, indicating a stable film even after exposure to pH 13 (see Figure 5b).

Likewise, the stability of acetylated surfaces (CH_acetyl) was tested. The results show that acidic (Figure 5c) solutions leave the film intact and negligible swelling occurs. In alkaline environments, also no significant changes in frequency and dissipation occur up to a pH value of 12 (Figure 5d). Similar to CH_neutral, pH 13 leads to some swelling of acetylated surfaces, but the dissipation or frequency shifts are marginal. Interestingly, however, a dry mass increase is observed for those films (the frequency shift is about −300 Hz, see Figure 5d), which can be attributed to the cleavage of ester or amide bonds and the formation and deposition of salts on the samples. The difference in the solubility of acetylated and neutralized surfaces could thus be used in the production of chitosan/chitinlike micropatterns employing soft- or UV-lithography as has been previously shown for cellulose thin films.^56^

In all, by testing the stability in a basic and acidic environment, valuable insights into the stability and interaction of the probed films with protons, water, and hydroxyl ions are obtained. The results gathered in Figure 5 can be applied in the functionalization of chitosan to adjust the coupling conditions for amino acids and peptides accordingly.

### 3.4 Chemical Composition of Chitosan Thin Films Coupled with N-Protected Amino Acids

**Figure 6:**
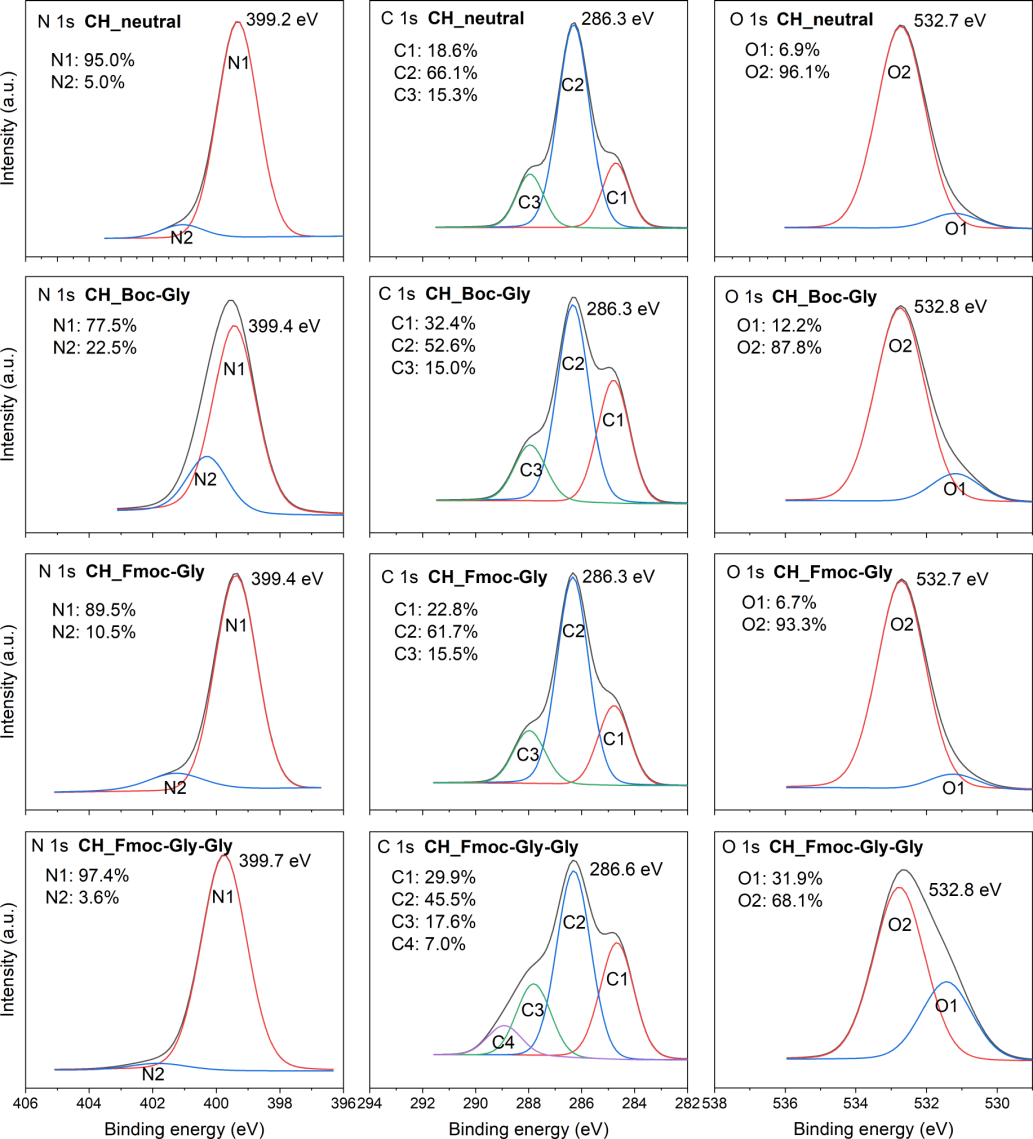
XPS high-energy resolution spectra N 1s, C 1s, and O 1s of neutralized chitosan (CH_neutral), Boc-Gly functionalized (CH_Boc-Gly), Fmoc-Gly functionalized (CH_Fmoc-Gly), and Fmoc-Gly-Gly functionalized (CH_Fmoc-Gly-Gly) chitosan thin-film samples. CH_Fmoc-Gly-Gly was obtained by acetylating remaining amino groups of CH_Fmoc-Gly, deprotection, and Fmoc-Gly coupling.

Surface elemental composition and binding modes of functionalized chitosan were compared to those of stable neutralized chitosan films, and the results are summarized in Figure 6. Theoretically calculated and measured surface elemental compositions are gathered in Table S2. A somehow lower O/C ratio than theoretically calculated was observed and can be explained by possible impurities (wide energy spectra Figure S3).

The C1 peak at 284.5 eV in C 1s spectra increases after the coupling from 17.9% to around 30% for Boc-Gly and for Fmoc-Gly-Gly surfaces, meaning that additional C–C bonds are now detectable. Those arise from the protective groups. The appearance of the C4 peak in the C 1s spectra of Fmoc-Gly-Gly at 288.9 eV, corresponds to the O=C–O bonds, confirming the formation of an ester through the acetyl capping before deprotection, and the presence of the Fmoc carbamate. A rise of O 1s from 6.9% to 12.2% after coupling of Boc-Gly, and to almost 32% after Fmoc-Gly-Gly coupling, also signifies the change on the surface.

The relative increase in O1 for 5.3% as well as the relative increase in C1 for 14.5% after coupling changed the ratio of C:O from 64:36 in pure chitosan to 67:33 in Boc coupled chitosan, more clearly showing a change in surface composition once functionalized. For Fmoc coupling, a relative increase in O1 peak was 23%, a 14% increase in C1, and the appearance of C4 peak suggested a more likely successful acetylation rather than immobilization of Fmoc-Gly-Gly. That is also why the C4 peak at 288.9 eV only appeared after the second Fmoc-Gly coupling, i.e., after capping, Fmoc deprotection, and second Fmoc-Gly coupling. Measurements of ToF-SIMS (Figure S2) also indicate the presence of Fmoc group as a peak at 179 *m*/*z* (C_14_H_10_^+^) only after second coupling and not also after first coupling.^57^ The XPS data for samples coupled only once with Fmoc-Gly (CH_Fmoc-Gly) do not vary much from XPS of neutralized chitosan surfaces (CH_neutral). The composition of Fmoc-Gly functionalized surface should differ greatly from Boc-Gly functionalized, but our results show a similar composition. Because of the presence of the base labile Fmoc group, a deprotection step with piperidine could be used prior to a second Fmoc-Gly coupling. The XPS data of Fmoc-Gly functionalized CH_neutral is similar to pristine CH_neutral indicating low coupling efficiency due to steric hindrance.

ATR-IR did not give any additional insights about the chemical surface composition change comparing neutralized chitosan sample to functionalized one.

### 3.5 Mass, Wetting, and Morphology of N-Protected Amino Acids

**Figure 7:**
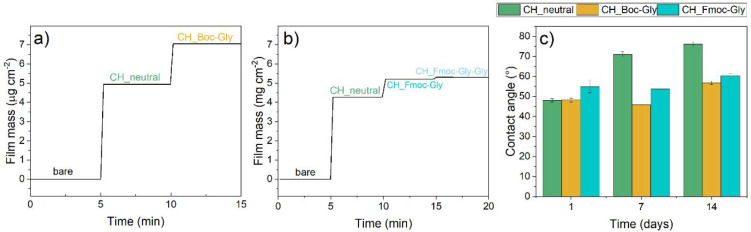
Stitched QCM-D data (in air) of bare substrate, neutralized chitosan (CH_neutral) surface, and chitosan coupled with (a) Boc-Gly (CH_Boc-Gly) or (b) Fmoc-Gly (CH_Fmoc-Gly) and Fmoc-Gly-Gly (CH_Fmoc-Gly-Gly) after acetylation and deprotection of Fmoc-Gly (coatings and derivatizations were performed in-between each QCM-D measurement step). (c) Water contact angle measurements of neutralized chitosan surface as well as Boc-Gly and Fmoc-Gly functionalized surface.

The dry masses in air after coating and coupling were assessed by QCM-D experiments. Starting with a CH_neutral film dry mass of 4.6 ± 0.35 μg/cm^2^ this increases to 6.2 ± 0.80 μg/cm^2^ after Boc-Gly coupling (Figure 7a). Unselective binding of reagents was tested in separate experiments, causing a 20% mass gain with only coupling reagent or with only Boc-Gly solution in water, showing that the CH_neutral films tend to adsorb these compounds. For Fmoc-Gly coupling, mass increased to 4.8 ± 0.59 μg/cm^2^ from an average base film mass of 4.6 ± 0.35 μg/cm^2^. This corresponds to a derivatization of 12% of all amine groups. For a full substitution of all amino groups with Fmoc-Gly, however, a final mass of 12.15 μg/cm^2^ should have been oberserved.

After capping by acetylation, Fmoc deprotection and subsequent Fmoc-Gly coupling, the overall mass increases to 4.9 ± 0.48 μg/cm^2^ from a base of 4.6 ± 0.35 μg/cm^2^ (Figure 7b). For 100% theoretical yield in all steps, however, the final film mass would be 14.05 μg/cm^2^. The lower coupled masses can have several explanations: (i) the primary amine group is not very accessible/reactive, (ii) EDC/HOBT are not very efficient coupling reagents, (iii) Fmoc as a relatively bulky protecting group can cause steric hindrance, (iv) during the chemical treatment some chitosan can detach, reducing the overall mass of the film despite or because of acetylation, and (v) deprotection is not efficient enough. Furthermore, the theoretical calculation considers the whole film mass, not only the film surface or sterically accessible points. Coupling efficiency of amino acids could be improved by changing the coupling reagent or by introducing spacers to overcome steric demands.

Even though substantial amounts of the protected amino acids were deposited in the first coupling step, subsequent pH dependent stability test in the QCM-D gave similar results as those shown in Figure 5a. This means that either insufficient amounts of nitrogen are derivatized into amides or that the Boc group was cleaved by exposure to low pH values with the former being more likely. To properly stabilize the films at a low pH value, one would need to acetylate the remainder primary amines as described in Section 3.3. This was performed after the first coupling of Fmoc-Gly to facilitate deprotection and the second coupling with another Fmoc-Gly molecule leading to an Fmoc-Gly-Gly peptide.

Interestingly, the wettability was expected to be higher once the samples were functionalized because the fluorene end group is a hydrophobic group also employed in the manufacture of superhydrophobic surfaces.^58^ Our measurements however only revealed a small difference in water contact angles between CH_neutral and their Boc and Fmoc modified counterparts at day one (Figure 7c). After storage in the dry, only minor changes in the contact angles are observed for modified surface which is similar to CH_acetyl films (Figure 3b). In preliminary studies, we have used isobutyl chloroformate as a coupling agent and found significantly higher contact angles of water, indicating the deposition of more Fmoc-gly. We therefore think that further studies for peptide binding on chitosan should include other coupling reagents than EDC/HOBT.

**Figure 8:**
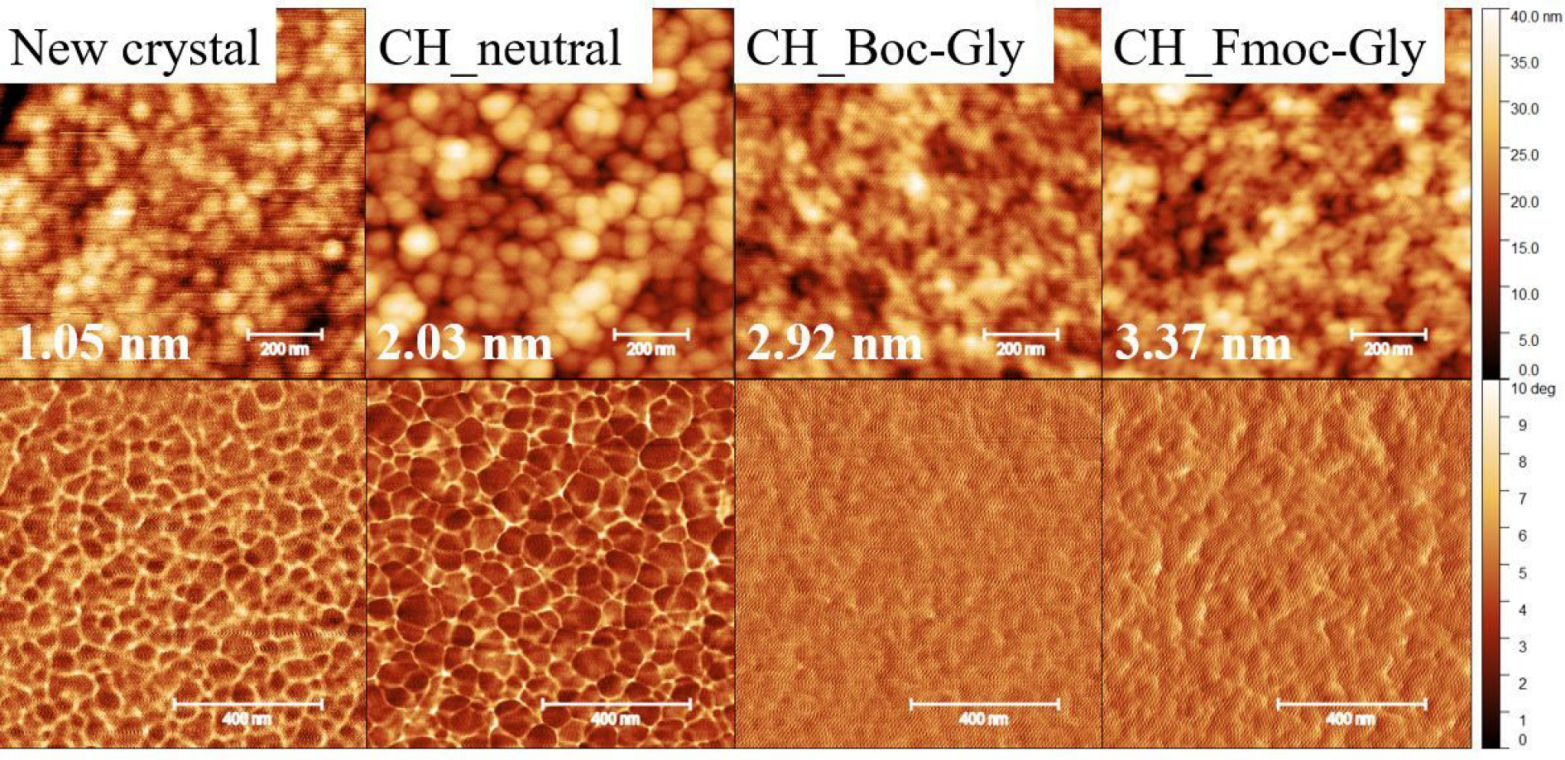
AFM images (1 × 1 μm)^2^. TOP row topography, BOTTOM row phase images: uncoated (New crystal), neutralized chitosan film (CH_neutral), Boc-Gly (CH_Boc-Gly), and Fmoc-Gly (CH_Fmoc-Gly) functionalized neutralized chitosan films on QCM-D gold-coated crystals. RMS roughness values are shown for the depicted height images.

AFM images in Figure 8 show a bare gold-coated QCM-D crystal next to CH_neutral and Boc-Gly and Fmoc-Gly functionalized surface. Functionalized surfaces have a higher RMS roughness. This could be due to different chemical treatments with water or DMSO. The treatment obviously exerts a certain swelling or desorption on the coating which however stay intact over the entire substrate.

## 4 Conclusions

Stable and smooth chitosan thin films can be prepared on silicon wafers and gold-coated QCM-D sensors by spin coating form aqueous hydrochloric acid solutions. Neutralization with 0.5 M aqueous sodium hydroxide solution and subsequent drying is necessary to deprotonate the primary amines and produce films stable in neutral water and DMSO as confirmed by XPS and QCM-D. During this neutralization, a certain film mass is removed, likely due to dissolution of the upper layers which are weaker bound. Nevertheless, the films stay intact on the substrate and can be used for basic interaction studies and several applications. An acetic anhydride pyridine mixture can be used to acetylate primary amines and likely also hydroxyls of the films, leading to a chitin mimetic surface. More investigations into the regioselectitivy, degree of acetylation, and crystallinity would be of interest to compare them with existing chitin films. Upon swelling, the water content of unmodified chitosan films was several times their dry mass but upon drying and aging, wetting substantially decreased. For chitin mimetics, water content was much lower and wetting stable over time. The chitinacous material is stable within a pH range of 13–2 which could make it useful as chemically resistant biopolymer coatings. However, pristine chitosan layers dissolve at pH 4 and substentially swell already at pH 6 as confirmed by QCM-D experiments. Boc and Fmoc protected amino acid can be grafted to chitosan layers by carbodiimide chemistry in water or DMSO with the former being more efficiently immobilized. This could be shown in detail by XPS and QCM-D dry mass data. Any chemical or solvent treatment of such thin films leads to roughening of the surfaces with the rougness remained in the nanometric range and the films being withouth major defects. The investigated materials and methods could form the basis for semisynthetic chitin, or with more sophisticated methods of chemical modifications peptidoglycans can be useful for biological interaction studies. Many other studies and applications as a biopolymer-based coating, including pattering or enzymatic degradation, can be anticipated.

## Supporting Information Available

Table S1, the composition of chitosan native, neutralized, and acetylated samples as determined from XPS survey spectra (CH_native, CH_neutral, CH_acetyl); Table S2, the composition of functionalized samples as determined from XPS survey spectra (CH_Boc-Gly, CH_Fmoc-Gly, CH_Fmoc-Gly-Gly). Figure S1, AFM images of chitosan samples exposed to different pH solutions; Figure S2, ToF-SIMS spectra of positive secondary ions from functionalized chitosan thin films (CH_Fmoc-Gly-Gly, CH_Fmoc-Gly, CH_acetyl); Figure S3, XPS survey spectra of chitosan, neutralized chitosan and functionalized chitosan thin films (PDF)

## References

[ref1] Vollmer W.; Blanot D.; De Pedro M. A. Peptidoglycan structure and architecture. FEMS Microbiol. Rev. 2008, 32 (2), 149–167. 10.1111/j.1574-6976.2007.00094.x. 18194336

[ref2] Turner R. D.; Mesnage S.; Hobbs J. K.; Foster S. J. Molecular imaging of glycan chains couples cell-wall polysaccharide architecture to bacterial cell morphology. Nat. Commun. 2018, 9 (1), 1263 10.1038/s41467-018-03551-y. 29593214 PMC5871751

[ref3] Carvalho L. C. R.; Queda F.; Almeida C. V.; Filipe S. R.; Marques M. M. B. From a Natural Polymer to Relevant NAG-NAM Precursors. Asian J. Org. Chem. 2018, 7 (12), 2544–2551. 10.1002/ajoc.201800592.

[ref4] Morimoto J.; Sarkar M.; Kenrick S.; Kodadek T. Dextran as a generally applicable multivalent scaffold for improving immunoglobulin-binding affinities of peptide and peptidomimetic ligands. Bioconjugate Chem. 2014, 25 (8), 1479–91. 10.1021/bc500226j. PMC4140544 25073654

[ref5] Kargl R.; Mohan T.; Köstler S.; Spirk S.; Doliška A.; Stana-Kleinschek K.; Ribitsch V. Functional Patterning of Biopolymer Thin Films Using Enzymes and Lithographic Methods. Adv. Funct. Mater. 2013, 23 (3), 308–315. 10.1002/adfm.201200607.

[ref6] Mohan T.; Nagaraj C.; Nagy B. M.; Bracic M.; Maver U.; Olschewski A.; Stana Kleinschek K.; Kargl R. Nano- and Micropatterned Polycaprolactone Cellulose Composite Surfaces with Tunable Protein Adsorption, Fibrin Clot Formation, and Endothelial Cellular Response. Biomacromolecules 2019, 20 (6), 2327–2337. 10.1021/acs.biomac.9b00304. 31070898 PMC6750646

[ref7] Kontturi E.; Spirk S. Ultrathin Films of Cellulose: A Materials Perspective. Front. Chem. 2019, 7, 488 10.3389/fchem.2019.00488. 31380342 PMC6652239

[ref8] Cheng J. C.; Pisano A. P. Photolithographic Process for Integration of the Biopolymer Chitosan Into Micro/Nanostructures. J. Microelectromech. Syst. 2008, 17 (2), 402–409. 10.1109/JMEMS.2008.916325.

[ref9] Wolf A. J.; Underhill D. M. Peptidoglycan recognition by the innate immune system. Nat. Rev. Immunol. 2018, 18 (4), 243–254. 10.1038/nri.2017.136. 29292393

[ref10] Abed M.; Towhid S. T.; Pakladok T.; Alesutan I.; Gotz F.; Gulbins E.; Lang F. Effect of bacterial peptidoglycan on erythrocyte death and adhesion to endothelial cells. Int. J. Med. Microbiol. 2013, 303 (4), 182–9. 10.1016/j.ijmm.2013.01.004. 23537625

[ref11] Tosoni G.; Conti M.; Diaz Heijtz R. Bacterial peptidoglycans as novel signaling molecules from microbiota to brain. Curr. Opin. Pharmacol. 2019, 48, 107–113. 10.1016/j.coph.2019.08.003. 31557694

[ref12] Murata J.-i.; Nagae H.; Ohya Y.; Ouchi T. Synthesis of muramyl dipeptide analogue—glucomannan conjugate and its stimulation activity against macrophage-like cells. Carbohydr. Polym. 1996, 29 (2), 111–118. 10.1016/0144-8617(96)00013-6.

[ref13] Liang H.; DeMeester K. E.; Hou C. W.; Parent M. A.; Caplan J. L.; Grimes C. L. Metabolic labelling of the carbohydrate core in bacterial peptidoglycan and its applications. Nat. Commun. 2017, 8, 15015 10.1038/ncomms15015. 28425464 PMC5411481

[ref14] Pranantyo D.; Xu L. Q.; Kang E.-T.; Chan-Park M. B. Chitosan-Based Peptidopolysaccharides as Cationic Antimicrobial Agents and Antibacterial Coatings. Biomacromolecules 2018, 19 (6), 2156–2165. 10.1021/acs.biomac.8b00270. 29672023

[ref15] Kittle J. D.; Wang C.; Qian C.; Zhang Y.; Zhang M.; Roman M.; Morris J. R.; Moore R. B.; Esker A. R. Ultrathin Chitin Films for Nanocomposites and Biosensors. Biomacromolecules 2012, 13 (3), 714 10.1021/bm201631r. 22263611

[ref16] Casteleijn M. G.; Richardson D.; Parkkila P.; Granqvist N.; Urtti A.; Viitala T. Spin coated chitin films for biosensors and its analysis are dependent on chitin-surface interactions. Colloids Surf., A 2018, 539, 261–272. 10.1016/j.colsurfa.2017.12.036.

[ref17] Elschner T.; Bračič M.; Mohan T.; Kargl R.; Stana Kleinschek K. Modification of cellulose thin films with lysine moieties: a promising approach to achieve antifouling performance. Cellulose 2018, 25 (1), 537–547. 10.1007/s10570-017-1538-9.

[ref18] Wu L.-Q.; Yi H.; Li S.; Rubloff G. W.; Bentley W. E.; Ghodssi R.; Payne G. F. Spatially Selective Deposition of a Reactive Polysaccharide Layer onto a Patterned Template. Langmuir 2003, 19 (3), 519–524. 10.1021/la026518t.

[ref19] Mohan T.; Kargl R.; Tradt K. E.; Kulterer M. R.; Bracic M.; Hribernik S.; Stana-Kleinschek K.; Ribitsch V. Antifouling coating of cellulose acetate thin films with polysaccharide multilayers. Carbohydr. Polym. 2015, 116, 149–58. 10.1016/j.carbpol.2014.04.068. 25458284

[ref20] Bračič M.; Mohan T.; Griesser T.; Stana-Kleinschek K.; Strnad S.; Fras-Zemljič L. One-Step Noncovalent Surface Functionalization of PDMS with Chitosan-Based Bioparticles and Their Protein-Repellent Properties. Adv. Mater. Interfaces 2017, 4 (21), 1700416 10.1002/admi.201700416.

[ref21] Fernandes R.; Wu L.-Q.; Chen T.; Yi H.; Rubloff G. W.; Ghodssi R.; Bentley W. E.; Payne G. F. Electrochemically Induced Depositions of a Polysaccharide Hydrogel onto a Patterned Surface. Langmuir 2003, 19, 4058 10.1021/la027052h.

[ref22] Jiang H.; Su W.; Caracci S.; Bunning T. J.; Cooper T.; Adams W. W. Optical waveguiding and morphology of chitosan thin films. J. Appl. Polym. Sci. 1996, 61 (7), 1163–1171. 10.1002/(SICI)1097-4628(19960815)61:7<1163::AID-APP12>3.0.CO;2-Z.

[ref23] Ligler F. S.; Lingerfelt B. M.; Price R. P.; Schoen P. E. Development of Uniform Chitosan Thin-Film Layers on Silicon Chips. Langmuir 2001, 17 (16), 5082–5084. 10.1021/la010148b.

[ref24] Carapeto A. P.; Ferraria A. M.; Botelho do Rego A. M. Chitosan Thin Films on Glass and Silicon Substrates. Microsc. Microanal. 2015, 21 (5), 13–4. 10.1017/S1431927615013872. 26227687

[ref25] Kumari S.; Tiyyagura H. R.; Pottathara Y. B.; Sadasivuni K. K.; Ponnamma D.; Douglas T. E. L.; Skirtach A. G.; Mohan M. K. Surface functionalization of chitosan as a coating material for orthopaedic applications: A comprehensive review. Carbohydr. Polym. 2021, 255, 117487 10.1016/j.carbpol.2020.117487. 33436247

[ref26] Abdullah J.; Ahmad M.; Karuppiah N.; Heng L. Y.; Sidek H. Immobilization of tyrosinase in chitosan film for an optical detection of phenol. Sens. Actuators, B 2006, 114 (2), 604–609. 10.1016/j.snb.2005.06.019.

[ref27] Baumgart T.; Offenhäusser A. Polysaccharide-Supported Planar Bilayer Lipid Model Membranes. Langmuir 2003, 19 (5), 1730–1737. 10.1021/la0261489.

[ref28] Murray C. A.; Dutcher J. R. Effect of Changes in Relative Humidity and Temperature on Ultrathin Chitosan Films. Biomacromolecules 2006, 7 (12), 3460–3465. 10.1021/bm060416q. 17154475

[ref29] Xu J.; McCarthy S. P.; Gross R. A.; Kaplan D. L. Chitosan Film Acylation and Effects on Biodegradability. Macromolecules 1996, 29 (10), 3436–3440. 10.1021/ma951638b.

[ref30] Kim D.-Y.; Nishiyama Y.; Kuga S. Surface acetylation of bacterial cellulose. Cellulose 2002, 9, 361–367. 10.1023/A:1021140726936.

[ref31] Kim H.; Tator C. H.; Shoichet M. S. Design of Protein-Releasing Chitosan Channels. Biotechnol. Prog. 2008, 24 (4), 932–937. 10.1021/bp070352a. 18324828

[ref32] Shamshina J. L.; Berton P.; Rogers R. D. Advances in Functional Chitin Materials: A Review. ACS Sustainable Chem. Eng. 2019, 7 (7), 6444–6457. 10.1021/acssuschemeng.8b06372.

[ref33] Neugebauer W.; Williams R. E.; Barbier J.-R.; Brzezinski R.; Willick G. Peptide synthesis on chitin. Int. J. Pept. Protein Res. 1996, 47 (4), 269–275. 10.1111/j.1399-3011.1996.tb01355.x. 8738652

[ref34] Meerovich I.; Smith D. D.; Dash A. K. Direct solid-phase peptide synthesis on chitosan microparticles for targeting tumor cells. J. Drug Delivery Sci. Technol. 2019, 54, 101288–101298. 10.1016/j.jddst.2019.101288.

[ref35] Costa F.; Maia S.; Gomes J.; Gomes P.; Martins M. C. L. Characterization of hLF1–11 immobilization onto chitosan ultrathin films, and its effects on antimicrobial activity. Acta Biomater. 2014, 10 (8), 3513–3521. 10.1016/j.actbio.2014.02.028. 24631659

[ref36] Monteiro C.; Fernandes H.; Oliveira D.; Vale N.; Barbosa M.; Gomes P.; MC L. M. AMP-Chitosan Coating with Bactericidal Activity in the Presence of Human Plasma Proteins. Molecules 2020, 25 (13), 1–10. 10.3390/molecules25133046. PMC7412451 32635294

[ref37] Lü X.; Zhang H.; Huang Y.; Zhang Y. A proteomics study to explore the role of adsorbed serum proteins for PC12 cell adhesion and growth on chitosan and collagen/chitosan surfaces. Regener. Biomater. 2018, 5 (5), 261–273. 10.1093/rb/rby017. PMC6184651 30338124

[ref38] Banta R. A.; Collins T. W.; Curley R.; O’Connell J.; Young P. W.; Holmes J. D.; Flynn E. J. Regulated phase separation in nanopatterned protein-polysaccharide thin films by spin coating. Colloids Surf., B 2020, 190, 110967 10.1016/j.colsurfb.2020.110967. 32199264

[ref39] Jia Y.; Peng Y.; Bai J.; Zhang X.; Cui Y.; Ning B.; Cui J.; Gao Z. Magnetic nanoparticle enhanced surface plasmon resonance sensor for estradiol analysis. Sens. Actuators, B 2018, 254, 629–635. 10.1016/j.snb.2017.07.061.

[ref40] Aydın E. B.; Aydın M.; Sezgintürk M. K. Electrochemical immunosensor based on chitosan/conductive carbon black composite modified disposable ITO electrode: An analytical platform for p53 detection. Biosens. Bioelectron. 2018, 121, 80–89. 10.1016/j.bios.2018.09.008. 30199712

[ref41] Barbosa M.; Costa F.; Monteiro C.; Duarte F.; Martins M. C. L.; Gomes P. Antimicrobial coatings prepared from Dhvar-5-click-grafted chitosan powders. Acta Biomater. 2019, 84, 242–256. 10.1016/j.actbio.2018.12.001. 30528610

[ref42] Bračič M.; Mohan T.; Kargl R.; Grießer T.; Heinze T.; Stana Kleinschek K. Protein repellent anti-coagulative mixed-charged cellulose derivative coatings. Carbohydr. Polym. 2021, 254, 117437 10.1016/j.carbpol.2020.117437. 33357910

[ref43] Takara E. A.; Marchese J.; Ochoa N. A. NaOH treatment of chitosan films: Impact on macromolecular structure and film properties. Carbohydr. Polym. 2015, 132, 25–30. 10.1016/j.carbpol.2015.05.077. 26256320

[ref44] Surface Analysis by Auger and X-Ray Photoelectron Spectroscopy; IM Publications: Chichester, UK, 2003; p 899.

[ref45] Moulder J. F.; Stickle W. F.; Sobol P. E.; Bomben K. D. Handbook of X-Ray Photoelectron Spectroscopy;Perkin-Elmer Corporation, 1995.

[ref46] Nečas D.; Klapetek P. Gwyddion: an open-source software for SPM data analysis. Cent Eur. J. Phys. 2012, 10 (1), 181–188. 10.2478/s11534-011-0096-2.

[ref47] Sauerbrey G. Verwendung von Schwingquarzen zur Wägung dünner Schichten und zur Mikrowägung. Eur. Phys. J. A 1959, 155 (2), 206–222. 10.1007/BF01337937.

[ref48] Kittle J. D.; Du X.; Jiang F.; Qian C.; Heinze T.; Roman M.; Esker A. R. Equilibrium water contents of cellulose films determined via solvent exchange and quartz crystal microbalance with dissipation monitoring. Biomacromolecules 2011, 12 (8), 2881–7. 10.1021/bm200352q. 21574564

[ref49] Kittle J. D.; Wang C.; Qian C.; Zhang Y.; Zhang M.; Roman M.; Morris J. R.; Moore R. B.; Esker A. R. Ultrathin chitin films for nanocomposites and biosensors. Biomacromolecules 2012, 13 (3), 714–8. 10.1021/bm201631r. 22263611

[ref50] Takara E. A.; Marchese J.; Ochoa N. A. NaOH treatment of chitosan films: Impact on macromolecular structure and film properties. Carbohydr. Polym. 2015, 132, 25–30. 10.1016/j.carbpol.2015.05.077. 26256320

[ref51] Kostov K.; Belamie E.; Alonso B.; Mineva T. Surface chemical states of cellulose, chitin and chitosan studied by density functional theory and high-resolution photoelectron spectroscopy. Bulg. Chem. Commun. 2018, 50, 135–147.

[ref52] Vesel A.; Mozetic M.; Strnad S.; Peršin Z.; Stana-Kleinschek K.; Hauptman N. Plasma modification of viscose textile. Vacuum 2009, 84 (1), 79–82. 10.1016/j.vacuum.2009.04.028.

[ref53] Almeida E. V. R.; Frollini E.; Castellan A.; Coma V. Chitosan, sisal cellulose, and biocomposite chitosan/sisal cellulose films prepared from thiourea/NaOH aqueous solution. Carbohydr. Polym. 2010, 80 (3), 655–664. 10.1016/j.carbpol.2009.10.039.

[ref54] Luo Y.; Pan X.; Ling Y.; Wang X.; Sun R. Facile fabrication of chitosan active film with xylan via direct immersion. Cellulose 2014, 21 (3), 1873–1883. 10.1007/s10570-013-0156-4.

[ref55] Mohan T.; Spirk S.; Kargl R.; Doliška A.; Vesel A.; Salzmann I.; Resel R.; Ribitsch V.; Stana-Kleinschek K. Exploring the rearrangement of amorphous cellulose model thin films upon heat treatment. Soft Matter 2012, 8 (38), 9807–9815. 10.1039/c2sm25911g.

[ref56] Wolfberger A.; Kargl R.; Griesser T.; Spirk S. Photoregeneration of trimethylsilyl cellulose as a tool for microstructuring ultrathin cellulose supports. Molecules 2014, 19 (10), 16266–16273. 10.3390/molecules191016266. 25310151 PMC6271022

[ref57] Enjalbal C.; Maux D.; Subra G.; Martinez J.; Combarieu R.; Aubagnac J.-L. Monitoring and quantification on solid support of a by-product formation during peptide synthesis by Tof-SIMS. Tetrahedron Lett. 1999, 40 (34), 6217–6220. 10.1016/S0040-4039(99)01158-2.

[ref58] Cao X.; Gao A.; Zhao N.; Yuan F.; Liu C.; Li R. Surfaces wettability and morphology modulation in a fluorene derivative self-assembly system. Appl. Surf. Sci. 2016, 368, 97–103. 10.1016/j.apsusc.2016.01.248.

