# Supplementary material for: Solid Phase Peptide Synthesis on Chitosan Thin Films
Source: Biomacromolecules. 2022 Jan 13;23(3):731–42. doi: 10.1021/acs.biomac.1c01155 (PMC8924862; doi:10.1021/acs.biomac.1c01155)
Supplement: Supplementary file 1 — bm1c01155_si_001.pdf [file bm1c01155_si_001.pdf]

Solid phase peptide synthesis on chitosan thin films

Tadeja Katan[†], Rupert Kargl[†], Tamilselvan Mohan[†], Tobias Alexander Steindorfer[†], Miran Mozetič[‡], Janez Kovač[‡], Karin Stana Kleinschek[†]

[†]*Graz University of Technology, Institute of Chemistry and Technology of Biobased Systems (IBioSys), Stremayrgasse 9, 8010 Graz, Austria*

[‡]*Department of Surface Engineering, Jožef Stefan Institute (IJS), Jamova 39, 1000 Ljubljana, Slovenia*

Table S1. Surface elemental composition of chitosan and neutralized chitosan thin film. Values are in at.%.

**Sample**	C	N	O	Na	Cl	**O/C ratio**
Chitin $_{theor.}$	57.1	7.1	35.7	0	0	0.63
CH_native	55.4±0.4	7.3±0.4	32.2±0.4	1.1	4.0±1.0	0.58
CH_neutral	58.9±0.4	7.3±0.4	33.6±0.4	0.1	0	0.57
CH_neutral $_{theor.}$	54.5	9.1	36.4	0	0	0.67
CH_acetyl	61.0±0.8	6.3±0.4	32.7±0.8	0	0	0.54
CH_acetyl $_{theor.}$	57.1	7.1	35.7	0	0	0.63

Table S1. Surface elemental composition of neutralized chitosan and functionalized chitosan thin film. Values are in at.%.

**Sample**	C	N	O	Other	**O/C ratio**

CH_neutral	58.9±0.4	7.3±0.4	33.6±0.4	0.10.05	0.57
CH_neutral theor.	54.5	9.1	36.4	0	0.67
CH_Boc-Gly	61.3±0.4	6.9±0.4	30.7±0.4	1.1±0.3	0.50
CH_Boc-Gly theor.	59.1	9.1	31.8	0	0.54
CH_Fmoc-Gly	61.6±0.4	7.6±0.4	30.9±0.4	0	0.50
CH_Fmoc-Gly theor.	71.9	6.2	21.9	0	0.30
CH_Fmoc-Gly-Gly	62.8±0.4	7.2±0.2	30.0±0.4	0	0.48
CH_Fmoc-Gly-Gly theor.	69.5	8.3	22.2	0	0.32

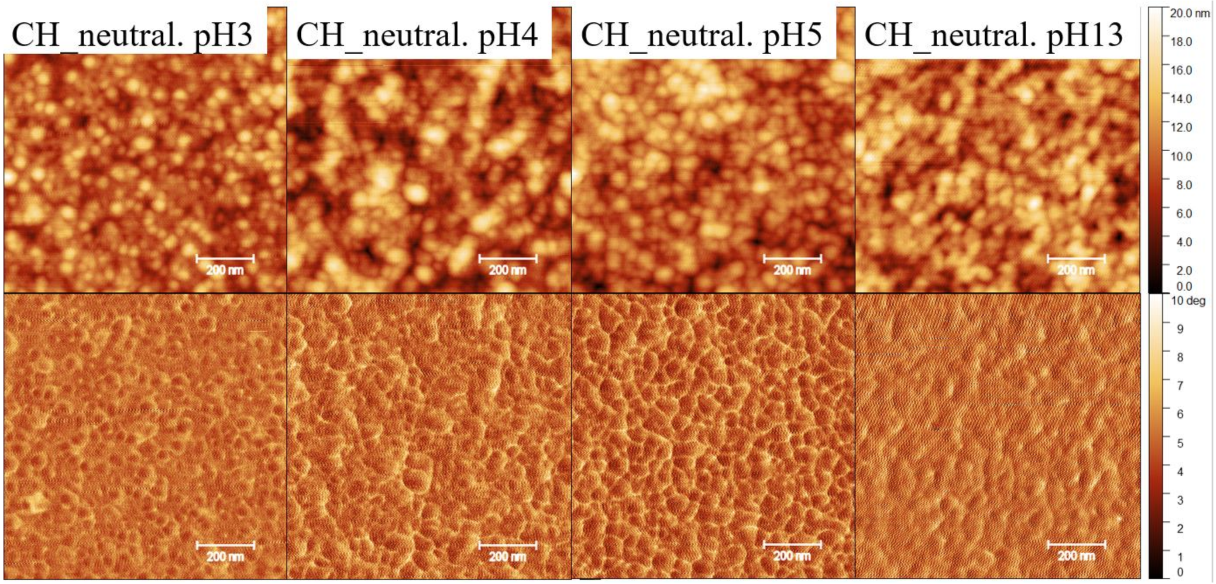

Figure S1. AFM images (1x1 µm)^2. TOP row topography, BOTTOM row phase images: neutralized chitosan films on QCM-D gold crystals at different pH exposures.

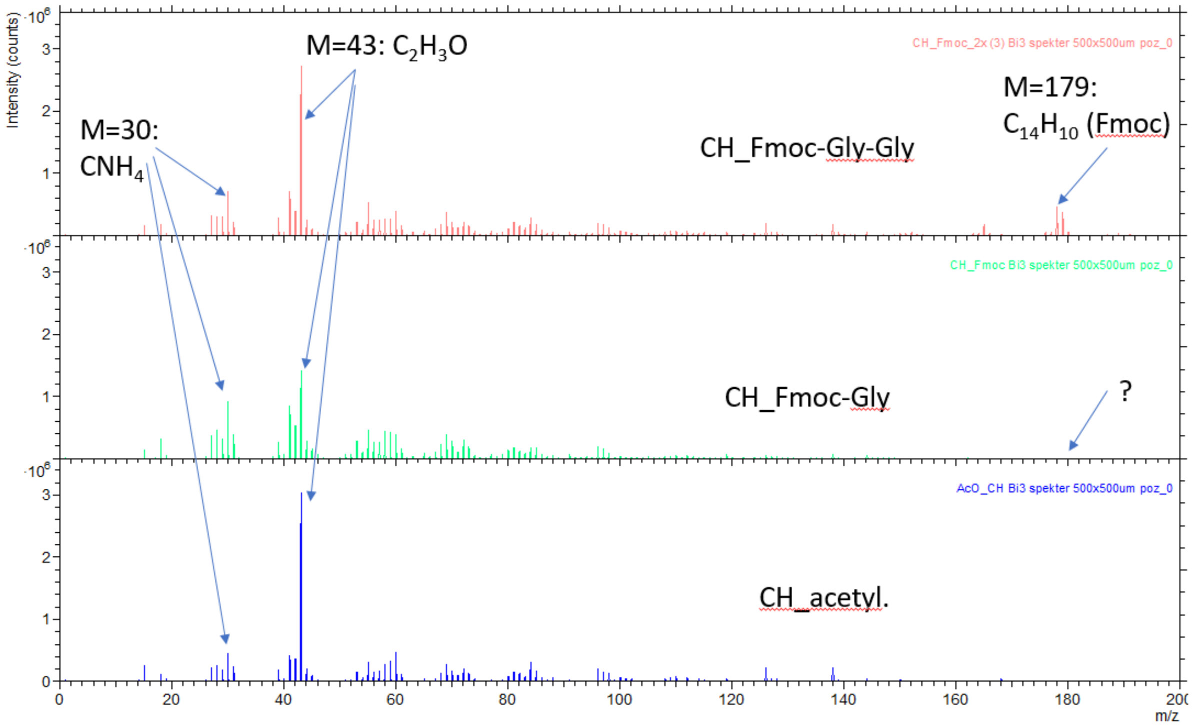

Figure S2. ToF-SIMS spectra of positive secondary ions from functionalized chitosan thin films (CH_Fmoc-Gly-Gly, CH_Fmoc-Gly, CH_acetyl)

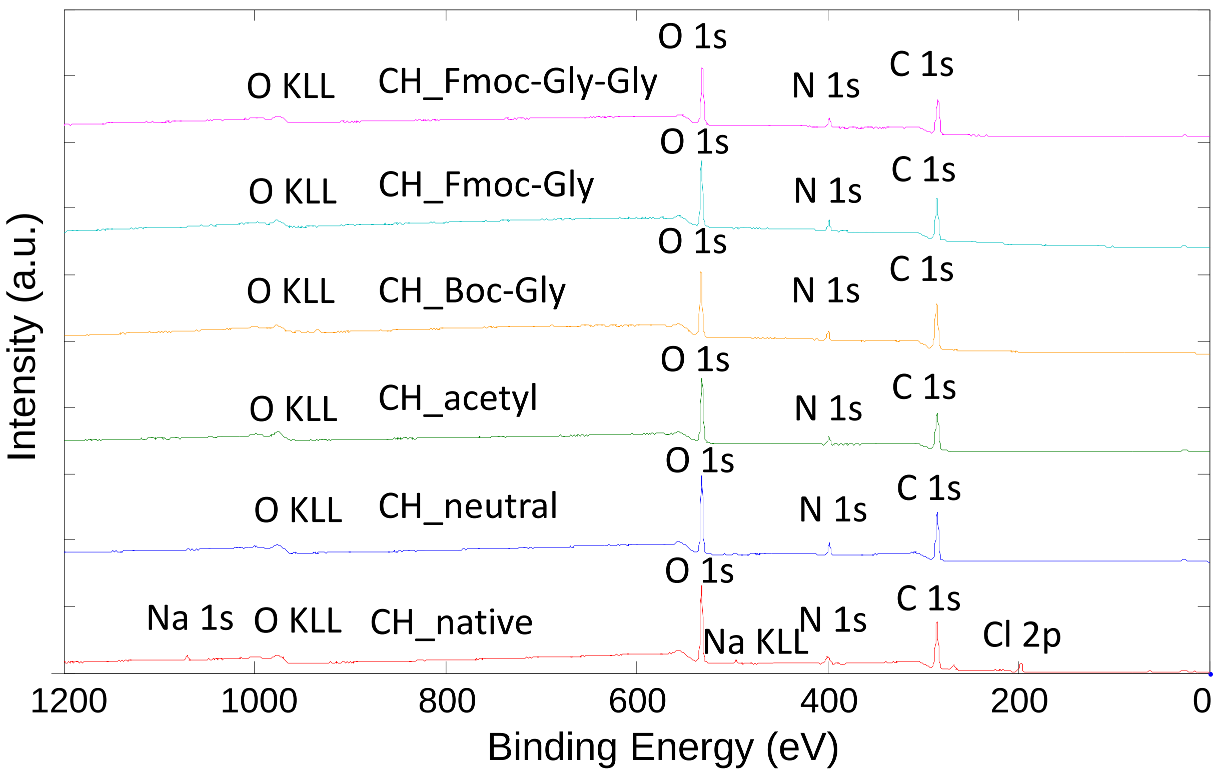

Figure S3. XPS survey spectra of chitosan, neutralized chitosan and functionalized chitosan thin films
